# Quantum Dynamics of Oblique Vibrational States in the Hénon–Heiles System

**DOI:** 10.1021/acs.jpca.3c03122

**Published:** 2023-10-06

**Authors:** José Zúñiga, Adolfo Bastida, Alberto Requena

**Affiliations:** Departamento de Química Física, Universidad de Murcia, 30100 Murcia, Spain

## Abstract

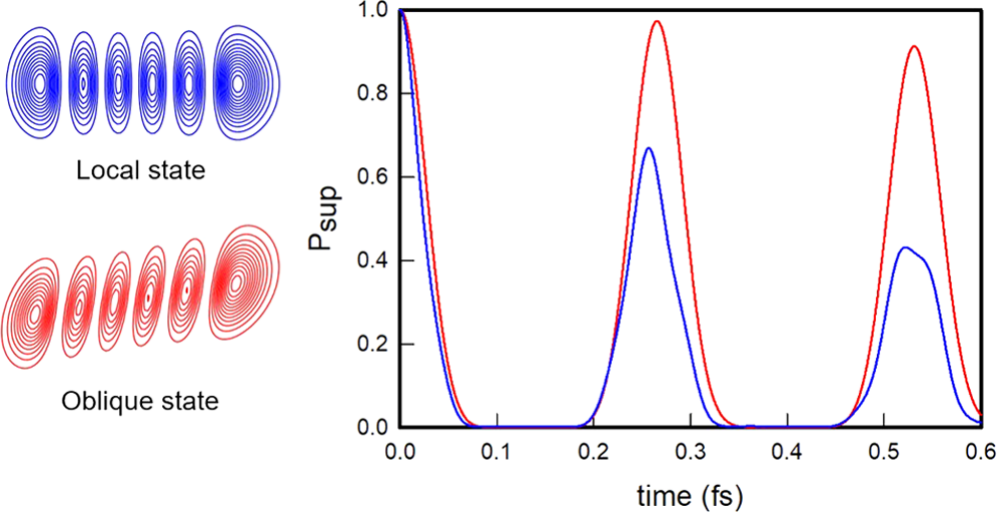

In this paper, we study the quantum time evolution of oblique nonstationary vibrational states in a Hénon–Heiles oscillator system with two dissociation channels, which models the stretching vibrational motions of triatomic molecules. The oblique nonstationary states we are interested in are the eigenfunctions of the anharmonic zero-order Hamiltonian operator resulting from the transformation to oblique coordinates, which are defined as those coming from nonorthogonal coordinate rotations that express the matrix representation of the second-order Hamiltonian in a block diagonal form characterized by the polyadic quantum number *n* = *n*_1_ + *n*_2_. The survival probabilities calculated show that the oblique nonstationary states evolve within their polyadic group with a high degree of coherence up to the dissociation limits on the short time scale. The degree of coherence is certainly much higher than that exhibited by the local nonstationary states extracted from the conventional orthogonal rotation of the original normal coordinates. We also show that energy exchange between the oblique vibrational modes occurs in a much more regular way than the exchange between the local modes.

## 1 Introduction

The time evolution of nonstationary quantum vibrational states in coupled oscillator systems is a topic of special interest in many areas of physical chemistry.^1−4^ This is fundamentally due to the great dynamical versatility that these states possess, stemming from the wide variety of temporal and spatial scales in which they are involved,^2,5^ which in turn manifests itself in a large number of vibrational energy exchange and transfer phenomena.^6−10^ From the theoretical point of view, the tracking of a nonstationary state is formally accomplished by solving the time-dependent Schrödinger equation,^4,11,12^ with computational challenges arising from the dimensionality of the system and the numerical instability of the time propagation, which are gradually being addressed as algorithms improves and computational power increases.^4^ Nevertheless, it is also useful to develop complementary dynamical theories of internal vibrational energy redistribution, capable of qualitatively and quantitatively capturing the essence of such processes.^6,8,10^

The vibrational dynamics of nonstationary quantum states depends on the system in which the state evolves, as well as on the way in which the state is initially prepared.^9,13^ Perhaps one of the cases where this fact is most clearly evidenced is in the widely studied vibrational normal-local mode duality of small molecules,^14−17^ where the initial preparation of a vibrationally excited local state and its subsequent time evolution may entail a possible coherent and selective energy transfer dynamics between the vibrational modes.^18−21^ Likewise, hyperspherical vibrational modes have been formulated, which extend their dynamics to the possible resonant state precursor of nonergodic unimolecular reactions.^22−24^ It is clear that the possible coherent time evolution of nonstationary vibrational states is conditioned by their degree of energetic excitation, as well as by the couplings they have with other states, which usually end up randomizing the energy transfer between the vibrational modes in the medium and high energy regimes. The question that arises, then, is to what extent a proper design of nonstationary vibrational states can effectively circumvent such couplings so that their dynamics become more coherent and extend over longer periods of time.

Normal coordinates are, by definition, those that eliminate the second-order kinetic and potential couplings of the Hamiltonian operator of the system.^25^ The transformation from normal to local coordinates reintroduces therefore these couplings.^14,15^ Generally, this transformation is linear and usually orthogonal, meaning that local coordinates are expressed as an orthogonal combination of normal coordinates. However, this linear combination is not the only one that can be used.^26−28^ Our group has been working for a long time on the possibility of performing generalized nonorthogonal linear transformations to describe the vibrational stretching motions of triatomic molecules,^29−32^ and we have recently focused on studying in detail this type of transformation that allows for the second-order Hamiltonian matrix of the system to be expressed in a block diagonal form, employing coupled oscillator models.^33−36^ The resulting coordinates can be visualized as individual nonorthogonal rotations of the original normal coordinates, that is, oblique coordinates.^36^

For systems of two coupled harmonic oscillators, oblique coordinates are constructed by decoupling the second-order Hamiltonian matrix blocks characterized by the polyadic quantum number *n* = *n*_1_ + *n*_2_. In a recent application^34^ of these coordinates to Hénon–Heiles systems with two dissociation channels, useful for modeling the stretching vibrational modes of triatomic molecules, we have shown that oblique coordinates are much better than normal and local coordinates when calculating the energy levels and wave functions of the system using the eigenfunctions of the corresponding uncoupled Hamiltonian operator as basis functions. In oblique coordinates, this set of basis functions is formed by the eigenfunctions of each uncoupled polyadic block, including anharmonicities. Accordingly, by initially preparing the system on one of these oblique basis function states, it is expected for it to evolve over time with a very low probability of leaving the initial polyadic block, meaning that the oblique nonstationary state will maintain a high degree of coherence within its polyadic block.

In this article, we compare the quantum dynamics of oblique nonstationary vibrational states to the dynamics of the corresponding local nonstationary states in a Hénon–Heiles system with two dissociation channels. This work adds, therefore, to the numerous dynamic studies to which the already seminal coupled Hénon–Heiles oscillator system has been subjected^37−52^ since its inception,^53^ by opening up the exploration of the quantum dynamics of oblique nonstationary vibrational states and the energy flows taking place among the oblique vibrational modes in coupled oscillator systems and in more realistic stretching motions of polyatomic molecules.

## 2 Theory

### 2.1 Coordinate Systems and Hamiltonian Operators

The Hamiltonian operator for the system of coupled Hénon–Heiles oscillators can be expressed as follows
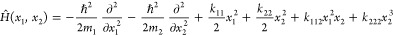
1where *x*_1_ and *x*_2_ are the coordinates of the system, *m*_1_ and *m*_2_ are the masses of the oscillators, *k*_11_ and *k*_22_ are the harmonic force constants, and *k*_112_ and *k*_222_ are the cubic coupling and anharmonicity constants. The natural harmonic frequencies of the uncoupled oscillators are therefore given as ω_1_ = (*k*_11_/*m*_1_)^1/2^ and ω_2_ = (*k*_22_/*m*_2_)^1/2^. Since this Hamiltonian operator contains no second-order coupling terms, coordinates *x*_1_ and *x*_2_ are, by definition, normal coordinates. To facilitate the coordinate transformations in this Hamiltonian operator, it is convenient to first introduce the mass-weighted coordinates *y*_1_ and *y*_2_ defined as follows
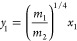
2
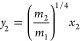
3The Hamiltonian operator thus becomes
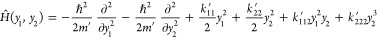
4where *m*′ = (*m*_1_*m*_2_)^1/2^ is a reduced mass, and the redefined force constants are given by the following expressions
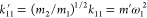
5
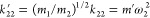
6
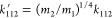
7
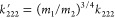
8The rotated coordinates *r*_1_ and *r*_2_, which are equivalent to the local coordinates in the context of molecular vibrations,^14,15^ are obtained by performing a 45° orthogonal rotation of coordinates *y*_1_ and *y*_2_ in the following form
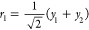
9
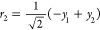
10The Hamiltonian operator is then written as follows
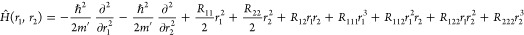
11with the rotated force constants given by
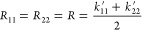
12
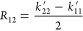
13
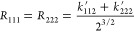
14
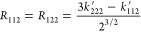
15

As discussed in our previous work on the Hénon–Heiles system,^34^ general oblique coordinates, *z*_1_ and *z*_2_, can be constructed by performing a nonorthogonal linear transformation of the rotated coordinates as follows
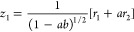
16
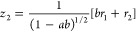
17where *a* and *b* are the transformation parameters. To preserve the symmetry of the energy potential function, the values of these parameters have to be equal (*a* = *b*), which simplifies the coordinate transformation as follows
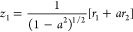
18
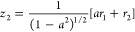
19The Hamiltonian operator in oblique coordinates *z*_1_ and *z*_2_ becomes then
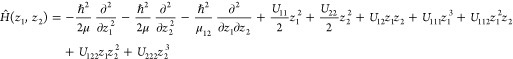
20with the reduced masses
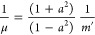
21
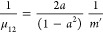
22and the second- and third-order force constants given by
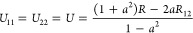
23
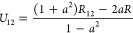
24
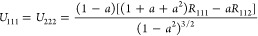
25
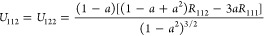
26

It is further convenient to redefine the parameter *a* in the angular form *a* = tan α, which allows us to write the nonorthogonal linear transformation in the following form
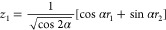
27
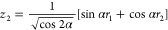
28This transformation accounts for a symmetric aperture of the *r*_1_ and *r*_2_ axes in angles −α and α, respectively, as shown in Figure 3 of our previous work.^34^

The specific oblique coordinates of interest, referred to as simply oblique coordinates, are defined such that the matrix representation of the second-order Hamiltonian operator of the system can be written in a block diagonal form^33,34^ characterized by the polyadic quantum number *n* = *n*_1_ + *n*_2_, where *n*_1_ and *n*_2_ are the quantum numbers corresponding to coordinates *z*_1_ and *z*_2_, respectively. For the Hénon–Heiles oscillator systems, we have previously demonstrated that the oblique rotation angle is given by^34^
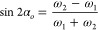
29meaning that when the uncoupled oscillators are degenerate (ω_1_ = ω_2_), the oblique coordinates coincide with the rotated coordinates (α_*o*_ = 0).

**Figure 1:**
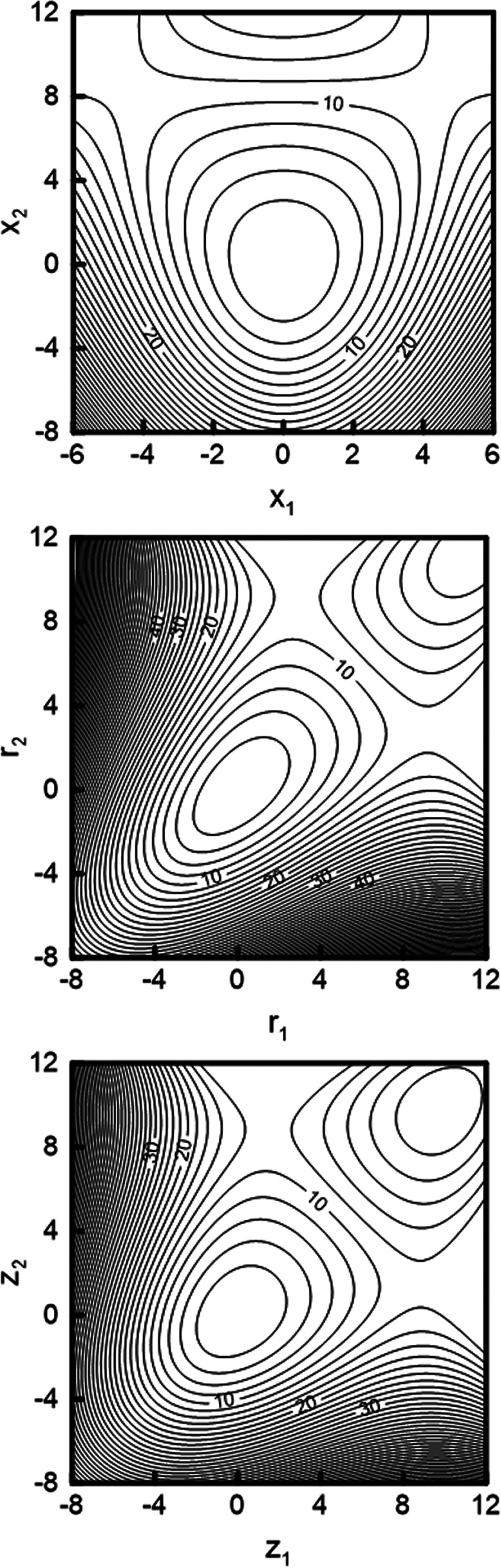
Contour plots of the Hénon–Heiles potential in normal (top panel), rotated (middle panel), and oblique (bottom panel) coordinates.

In our previous work,^34^ we have shown that the use of oblique coordinates for Hénon–Heiles systems with two dissociation channels, which mimic the behavior of the stretching modes of triatomic molecules, provides much faster variational convergence of the energy levels and eigenfunctions of the system than normal and rotated coordinates.^34^ Accordingly, to study the time evolution of nonstationary quantum states expressed in oblique coordinates, we have used the Hénon–Heiles system with masses *m*_1_ = *m*_2_ = 1, harmonic frequencies ω_1_ = 1.3 and ω_2_ = 0.7, and third-order force constants *k*_112_ = −0.1 and *k*_222_ = −0.01, in atomic units (ℏ = 1). This potential was introduced by Eastes and Marcus^54^ to develop semiclassical methods of calculating bound states of coupled systems^54−58^ and has also been used to study the propagation of Gaussian wave packets.^37,44,46,47,51^ The potential has a dissociation energy of *E*_dis_ = 11.4601 au and a total number of 83 bound states, which essentially have a regular, nonergodic structure.^37,58^ The values of the parameters for this Hénon–Heiles system in normal, rotated, and oblique coordinates are given in Table 1, and the contour plots of the potential energy function in the three coordinate systems are shown in Figure 1.

**Table 1:** Parameters of the Hénon–Heiles System in Different Coordinate Systems

normal	rotated	oblique
*m*_1_ = 1.0	*m*′ = 1.0	μ = 0.9539
*m*_2_ = 1.0		μ_12_ = −3.1798
ω_1_ = 1.3	ω_*r*,1_ = 1.044	ω_*o*,1_ = 1.0
ω_2_ = 0.7	ω_*r*,2_ = 1.044	ω_*o*,2_ = 1.0
*k*_11_ = 1.69	R = 1.09	*U* = 0.9539
*k*_22_ = 0.49		
*k*_12_ = 0	*R*_12_ = −0.6	*U*_12_ = −0.2862
*k*_222_ = −0.01	*R*_111_ = −0.0389	*U*_111_ = −0.03591
*k*_112_ = −0.1	*R*_112_ = 0.0247	*U*_112_ = 0.01341
α_*n*_ = 0°	α_*r*_ = 45°	α*_o_* = −8.73°

### 2.2 Stationary States

The time-dependent quantum dynamics of the Hénon–Heiles system has been studied using the representation of the stationary states, which are given by the general expression
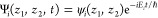
30where ψ_*i*_(*z*_1_, *z*_2_) and *E*_*i*_ are the eigenfunctions and eigenvalues of the Hamiltonian operator, respectively, i.e., the solutions of the time-independent Schrödinger equation.
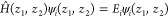
31This eigenvalue equation is solved by writing the Hamiltonian operator (eq 20) in the following form
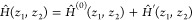
32where *Ĥ*^(0)^(*z*_1_, *z*_2_) is the zero-order Hamiltonian of the system given by the sum of the individual anharmonic Hamiltonian of each coordinate, i.e.
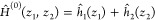
33where
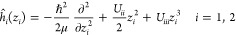
34and *Ĥ*′(*z*_1_, *z*_2_) is the operator containing the second- and third-order couplings.
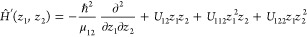
35

The eigenvalue equation for the zero-order Hamiltonian operator is then given by
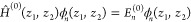
36where the eigenfunctions ϕ_*n*_(*z*_1_, *z*_2_) are the products of one-dimensional functions ϕ_*n*_1__(*z*_1_) and ϕ_*n*_2__(*z*_2_), that is,
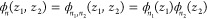
37which are the solutions of the eigenvalue equations
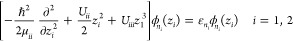
38solved variationally by expressing the eigenfunctions ϕ_*n*_*i*__(*z*_*i*_) as linear combinations of harmonic functions φ_*v*_*i*__(*z*_*i*_) in the following form.
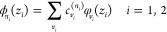
39Further diagonalization of the resulting Hamiltonian matrix provides eigenvector coefficients *c*_*v_i_*_^(*n*_*i*_)^ and eigenenergies ε_*n*_*i*__. The energy eigenvalues of the zero-order Hamiltonian *E*_*n*_^(0)^ are then just the sums of the energies of the individual anharmonic oscillators, i.e.
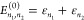
40

The eigenfunctions of the zero-order system, ϕ_*n*_1_,*n*_2__(*z*_1_, *z*_2_), are next used as basis functions to determine, also variationally, the energy levels and eigenfunctions of the complete system. Specifically, the eigenfunctions ψ_*i*_(*z*_1_, *z*_2_) of the complete system are expressed as linear combinations of these basis functions as follows
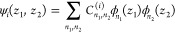
41The Hamiltonian matrix for the complete system is then diagonalized, yielding the energy eigenvalues *E*_*i*_ and coefficients *C*_*n*_1_,*n*_2__^(*i*)^ of the eigenfunctions of the Hénon–Heiles system in the nonorthogonal linear coordinates *z*_1_ and *z*_2_, which include the normal, rotated, and oblique coordinates as particular cases.

We have computed variationally the energy levels and eigenfunctions of the Hénon–Heiles potential in the three different coordinate systems. In all three cases, we selected the anharmonic basis functions as those belonging to successive polyadic blocks characterized by the quantum number *n* = *n*_1_ + *n*_2_. To achieve convergence of all bound energy levels of the potential to the first five significant figures in both normal and rotated coordinates, it was necessary to use all of the basis functions up to the polyadic block *n* = 40, resulting in a total of 861 functions. However, for the more favorable oblique coordinates,^34^ it was sufficient to use the basis functions comprised up to the polyadic block *n* = 23, which resulted in a total of solely 300 functions.

**Figure 2:**
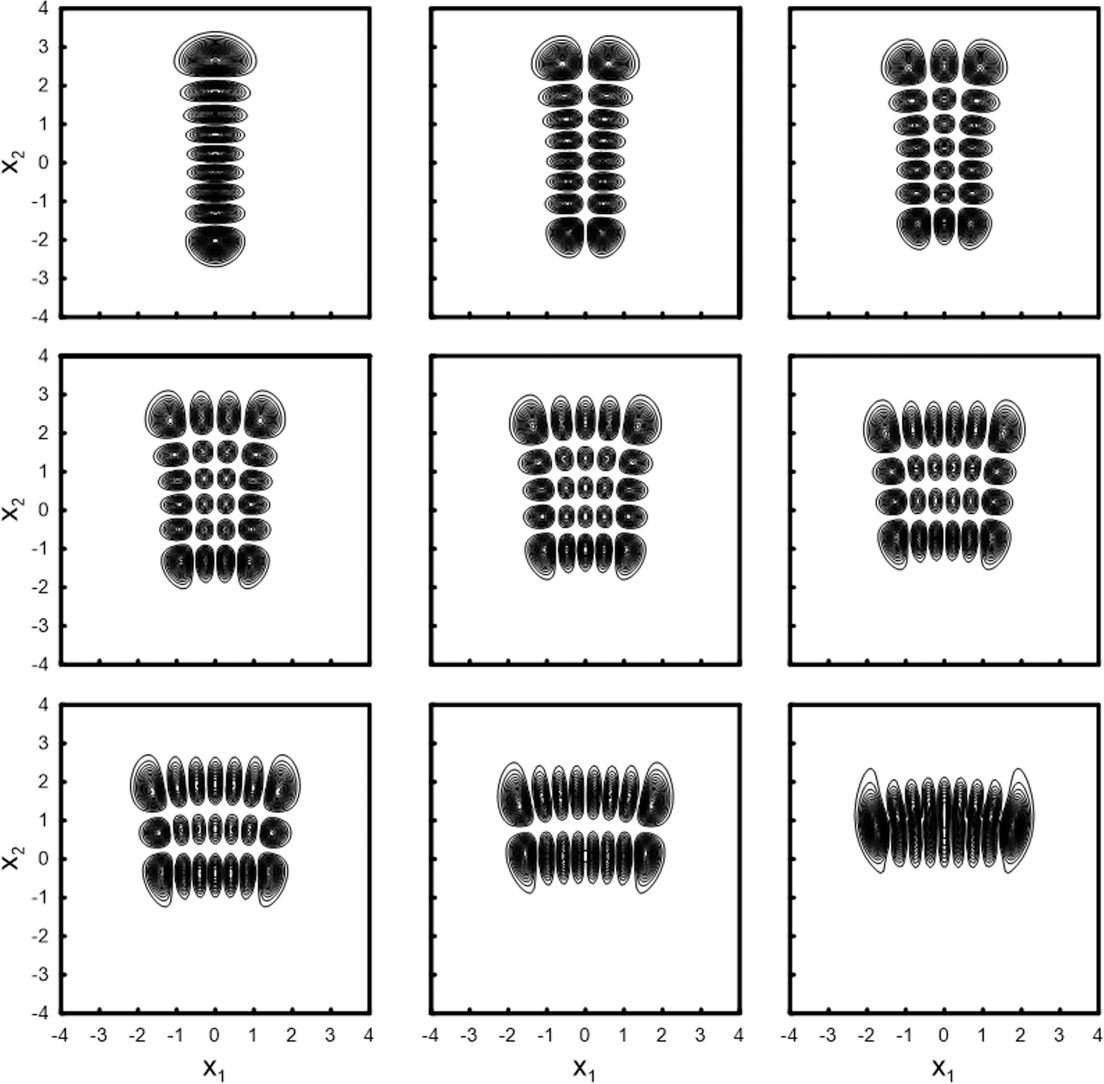
Variational wave functions of the *n* = 8 polyadic block in normal coordinates.

In Table 2, we include the variationally calculated energy levels for the Hénon–Heiles potential, sorted by polyadic groups, and their assignments made using the three coordinate systems. The calculated energy levels agree well with those obtained by Swimm and Delos^58^ using a set of harmonic basis functions. For the normal coordinates, we give the dominant squared coefficients of the eigenfunctions, whose values decrease as the energy excitation increases until they are no longer reliable for assigning energy levels with normal quantum numbers. Levels misassigned in this way begin to appear in increasing numbers from the *n* = 6 polyadic block upward. However, this does not mean that these levels cannot be assigned well in normal coordinates. In fact, all eigenfunctions of the system maintain a regular structure practically up to the dissociation limit, as shown in Figure 2, where the eigenfunctions belonging to the *n* = 8 polyadic block are depicted. With the sole exception of the last bound state, 83, all states of this Hénon–Heiles potential can be assigned with no problems by visual inspection of their eigenfunctions, confirming the nonergodic character of the potential as revealed long ago by means of classical and semiclassical treatments.^37,58^ The limitations of normal coordinates for assigning bound states using the dominant coefficients of the eigenfunctions are due to the curvilinear profiles that the nodal lines of the eigenfunctions adopt as the energy excitation increases, which cannot be reproduced by the rectilinear normal coordinates used in this work.

**Table 2:** Energy Levels and Polyaddition Probabilities of the Hénon–Heiles System^a^

				normal	rotated	oblique
*i*	*n*^(a)^	*n*_1_, *n*_2_	*E*	|*C*_*n*_1_,*n*_2__|_max_^2^	*P*_*i*_(*n*)	*P*_*i*_(*n*)
1	0	0,0	0.9955	0.9975	0.9733	0.9998
2	1	0,1	1.6870	0.9920	0.9321	0.9992
3	1	1,0	2.2781	0.9786	0.9545	0.9973
4	2	0,2	2.3750	0.9861	0.8553	0.9989
5	2	1,1	2.9584	0.9340	0.8987	0.9916
7	2	2,0	3.5479	0.9396	0.9166	0.9914
6	3	0,3	3.0596	0.9797	0.7499	0.9986
8	3	1,2	3.6347	0.8876	0.8078	0.9871
10	3	2,1	4.2162	0.8199	0.8356	0.9736
13	3	3,0	4.8043	0.8803	0.8600	0.9815
9	4	0,4	3.7404	0.9727	0.6255	0.9984
11	4	1,3	4.3069	0.8393	0.6910	0.9835
14	4	2,2	4.8799	0.7038	0.7239	0.9581
17	4	3,1	5.4597	0.6630	0.7458	0.9442
21	4	4,0	6.0463	0.8046	0.7862	0.9679
12	5	0,5	4.4176	0.9650	0.4926	0.9978
15	5	1,4	4.9749	0.7891	0.5598	0.9808
18	5	2,3	5.5390	0.5918	0.5941	0.9442
22	5	3,2	6.1099	0.4716	0.6112	0.9100
26	5	4,1	6.6878	0.4772	0.6357	0.9017
31	5	5,0	7.2730	0.7093	0.6983	0.9471
16	6	0,6	5.0908	0.9564	0.3622	0.9967
19	6	1,5	5.6385	0.7364	0.4261	0.9789
23	6	2,4	6.1931	0.4847	0.4596	0.9320
27	6	3,3	6.7546	0.3130	0.4723	0.8787
32	6	4,2	7.3234		0.4826	0.8416
37	6	5,1	7.8996		0.5149	0.8447
43	6	6,0	8.4835	0.6043	0.6008	0.9206
20	7	0,7	5.7601	0.9468		0.9948
24	7	1,6	6.2975	0.6813	0.3010	0.9773
28	7	2,5	6.8419	0.3837	0.3324	0.9210
33	7	3,4	7.3935		0.3425	0.8497
38	7	4,3	7.9524		0.3442	0.7865
44	7	5,2	8.5190		0.3547	0.7524
50	7	6,1	9.0934		0.3956	0.7720
57	7	7,0	9.6762	0.4923	0.4998	0.8858
25	8	0,8	6.4253	0.9360		0.9915
29	8	1,7	6.9515	0.6234		0.9758
34	8	2,6	7.4850		0.2939	0.9109
39	8	3,5	8.0259		0.2314	0.8225
45	8	4,4	8.5743		0.2301	0.7353
51	8	5,3	9.1305		0.2304	0.6694
58	8	6,2	9.6948		0.2855	0.6424

				normal	rotated	oblique
*i*	*n*^(a)^	*n*_1_, *n*_2_	*E*	|*C*_*n*_1_,*n*_2__|_max_^2^	*P*_*i*_(*n*)	*P*_*i*_(*n*)
64	8	7,1	10.2676			0.6793
73	8	8,0	10.8493	0.3680		0.8119
30	9	0,9	7.0861	0.9236		0.9863
35	9	1,8	7.6003	0.5627		0.9737
40	9	2,7	8.1220			0.9007
46	9	3,6	8.6513			0.7960
52	9	4,5	9.1882			0.6869
59	9	5,4	9.7331			0.5936
65	9	6,3	10.2861			0.5303
72	9	7,2	10.8478			0.5047
82	9	8,1	11.4158			0.3992
36	10	0,10	7.7423	0.9090		0.9796
41	10	1,9	8.2435	0.4988		0.9699
47	10	2,8	8.7524			0.8891
53	10	3,7	9.2689			0.7686
60	10	4,6	9.7932			0.6391
67	10	5,5	10.3253			0.5213
74	10	6,4	10.8657			0.4149
81	10	7,3	11.4129			0.3536
42	11	0,11	8.3939	0.8922		0.9673
48	11	1,10	8.8805	0.4317		0.9631
54	11	2,9	9.3752			0.8739
61	11	3,8	9.8776			0.7369
68	11	4,7	10.3877			0.5877
75	11	5,6	10.9053			0.4445
83	11	6,5	11.4323			0.2282
49	12	0,12	9.0403	0.8705		0.9509
55	12	1,11	9.5108	0.3641		0.9507
62	12	2,10	9.9895			0.8506
69	12	3,9	10.4759			0.6954
77	12	4,8	10.9702			0.5121
57	13	0,13	9.6813	0.8434		0.9275
63	13	1,12	10.1332	0.2876		0.9245
70	13	2,11	10.5938			0.7747
78	13	3,10	11.0612			0.5051
66	14	0,14	10.3166	0.8060		0.8924
71	14	1,13	10.7474			0.8582
79	14	2,12	11.1862			0.5727
76	15	0,15	10.9465	0.7252		0.7976
80	15	1,15	11.3543			0.6903

a *n* = *n*_1_ + *n*_2_.

When using rotated and oblique coordinates, direct assignments by the dominant coefficients of the wave functions are no longer feasible, since the basis functions do not mimic the exact eigenfunctions of the potential well.^17,32^ For these coordinates, it makes more sense to analyze to what extent the variational wave functions are composed of contributions from the basis functions belonging to the same polyad. These polyadic contributions are quantified using the polyadic probability *P*_*i*_(*n*), which is defined as follows
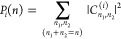
42

**Figure 3:**
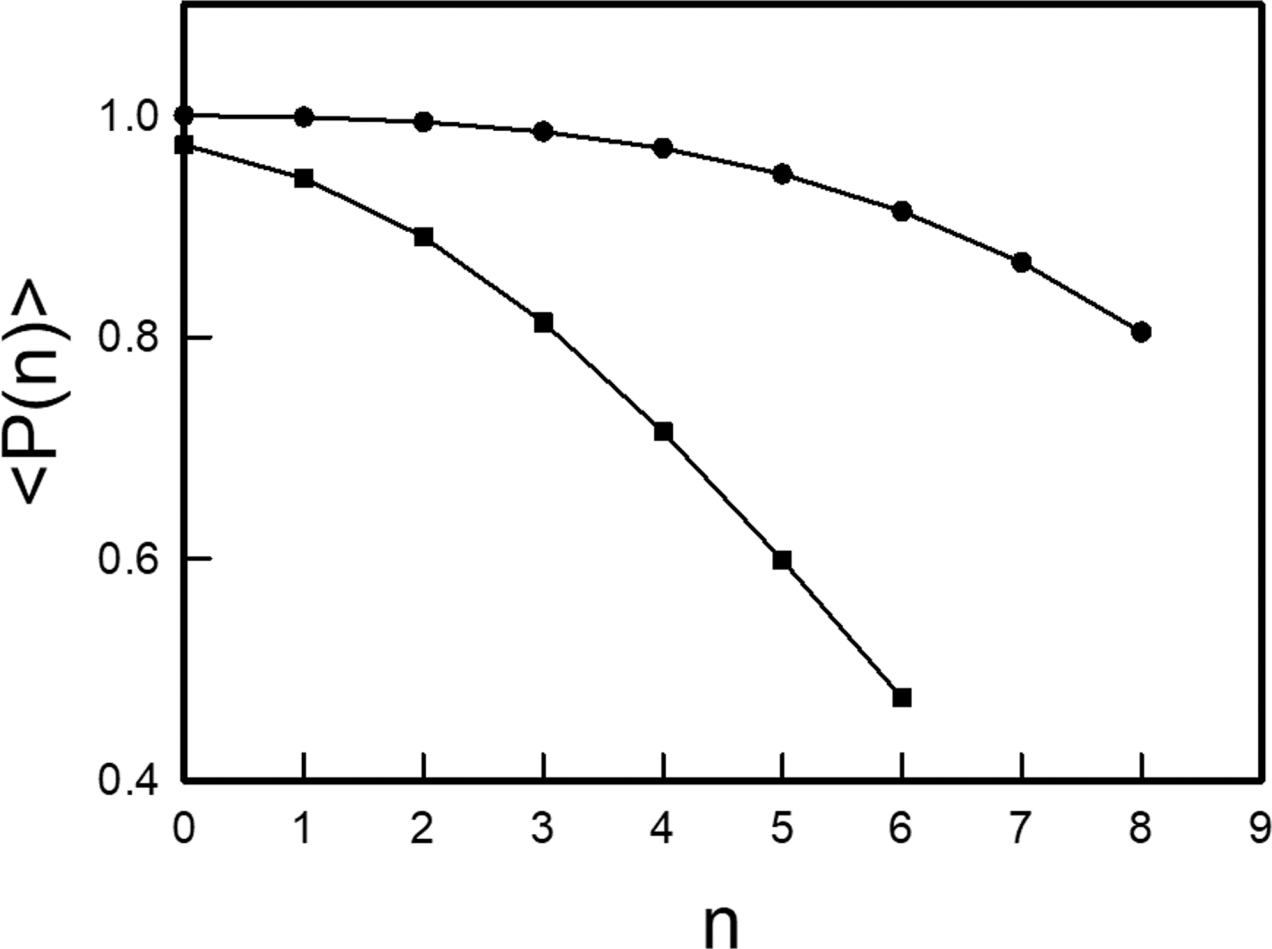
Average polyadic probabilities of the states belonging to the polyadic groups of the Hénon–Heiles potential in rotated (squares) and oblique (circles) coordinates.

Table 2 includes the polyadic probabilities of the vibrational states of the Hénon–Heiles potential calculated using rotated and oblique coordinates. As observed, the oblique polyadic probabilities are always larger than the rotated polyadic probabilities. In fact, the oblique polyadic probabilities allow us to assign the polyadic blocks of all of the bound states of the potential, while the rotated coordinates become less useful as energy increases and are no longer reliable from the polyadic block *n* = 7 upward, as shown in Figure 3, where the average values of the polyadic probabilities provided by both coordinate systems for each polyadic group *n* are plotted.

## 3 Results and Discussion

### 3.1 Time Evolution of Nonstationary States

Let us now consider the study of the quantum dynamics of nonstationary states in the Hénon–Heiles oscillator. Suppose that the system is initially prepared, at time *t* = 0, in a given nonstationary state Ψ(*z*_1_, *z*_2_, *t* = 0) ≡ Ψ(*z*_1_, *z*_2_, 0). The wave function describing the system at any other instant of time can then be written as a linear combination of stationary states in the following form
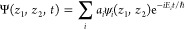
43where the coefficients *a*_*i*_ are given by
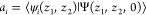
44The probability for the system to stay in the initial nonstationary state, i.e., the survival probability, is then given by
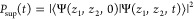
45which, by using eq 43, can be developed as follows
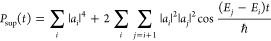
46

As we saw in the previous section, the use of oblique coordinates provides variational wave functions for the Hénon–Heiles system that can be represented, to a large extent, as linear combinations of the polyadic basis functions to which the variational eigenfunction belongs. From a dynamical point of view, this means that if the system is initially prepared in one of the nonstationary oblique basis functions, it is expected to evolve mostly within the polyadic block of the initial state, with a relatively low probability of leaving this block.

Let us consider then that the initial state is one of the eigenstates ϕ_*m*_1__(*z*_1_)ϕ_*m*_2__(*z*_2_) of the oblique zero-order system, i.e.
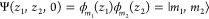
47This state contains *m*_1_ quanta of vibrational energy in the oblique anharmonic mode *z*_1_ and *m*_2_ quanta in the oblique anharmonic mode *z*_2_. By substituting eqs 41 and 47 into eq 44, it is easy to verify that the coefficients *a*_*i*_ of the time-dependent wave function (eq 43) are given by
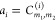
48The initial wave function can be then expressed as follows
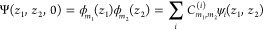
49and the time-dependent wave function and the survival probability become
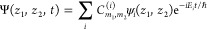
50
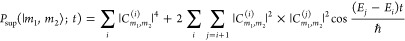
51

We can also compute the probability of the system undergoing a transition from the initial nonstationary state |*m*_1_, *m*_2_⟩ to any other nonstationary state |*m*_1_^′^, *m*_2_^′^⟩. This transition probability is defined as
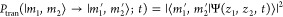
52and using again eqs 41 and 47, the transition probability becomes
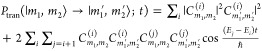
53

Furthermore, the probability that the initial nonstationary state |*m*_1_, *m*_2_⟩ remains in its polyadic block *m* = *m*_1_ + *m*_2_ is equal to the sum of the survival probability and the transition probabilities to the remaining states of the polyadic block, i.e.
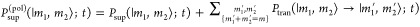
54This is the so-called polyadic survival probability of the nonstationary state |*m*_1_, *m*_2_⟩.

As far as the energies of the nonstationary states of the system are concerned, since the system is conservative, that is, the Hamiltonian operator is time-independent, the total energy remains constant and is given by
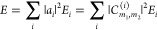
55In contrast, the energies of the anharmonic vibrational modes *z*_1_ and *z*_2_ do vary with time. The Hamiltonian operators that account for these energies are *ĥ*_1_(*z*_1_) and *ĥ*_2_(*z*_2_), so we can quantify the energy contents of the vibrational modes, *E*_1_(*t*) and *E*_2_(*t*), as the expected values of the individual Hamiltonian operators, that is^20,40^
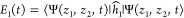
56
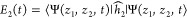
57Using eq 50 for the time-dependent wave function, we obtain
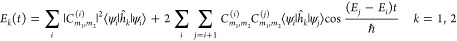
58where
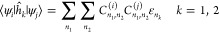
59

Let us begin by analyzing the time evolution of the nonstationary states in the polyadic block *m* = 1. This block is composed of the basis functions ϕ_1_(*z*_1_) ϕ_0_(*z*_2_) ≡ |1,0⟩ and ϕ_0_(*z*_1_) ϕ_1_(*z*_2_) ≡ |0, 1⟩. When chosen as oblique initial states, these basis functions are given by the following expansions
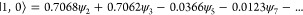
60
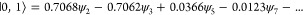
61
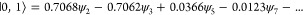
with energies *E*_|1,0⟩_ = *E*_|0,1⟩_ = 1.9839, and when chosen as rotated initial states, which we will hereafter refer to as local states, they are given by the following expansions
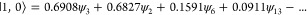
62
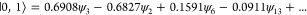
63with energies *E*_∥1,0⟩_ = *E*_|0,1⟩_ = 2.0736. As we can see, the dominant coefficients in both sets of coordinates correspond to the eigenfunctions ψ_2_ and ψ_3_ of the Hénon–Heiles system, which contribute the most to the *m* = 1 polyadic block (see Table 2). Moreover, the coefficients for the oblique states have absolute values larger than those for the local states. Therefore, the oblique initial nonstationary states include larger contributions from the polyadic variational functions ψ_2_ and ψ_3_ than those from their local initial nonstationary states. So, let us see what impact this has on the time evolution of the nonstationary states.

**Figure 4:**
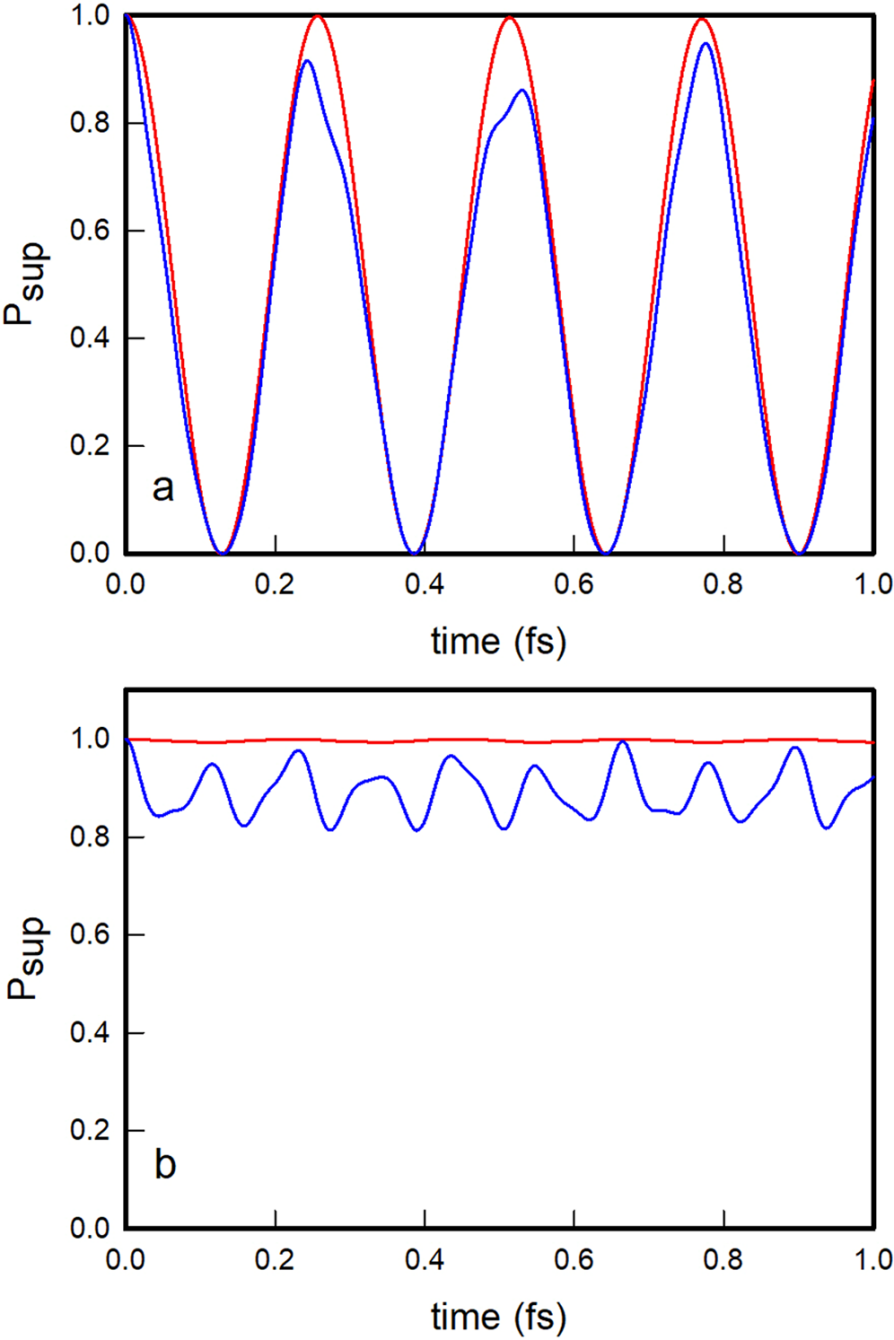
Polyadic (a) and polyadic survival (b) probabilities for the oblique (red) and local (blue) |1, 0⟩ nonstationary states.

In Figure 4a, we plot the survival probabilities of the oblique and local |1, 0⟩ nonstationary states as a function of time. As observed, the oblique |1, 0⟩ nonstationary state evolves by displaying quasi-periodic oscillations with recurrent probability maxima that practically reach unity, while the local |1, 0⟩ state shows more irregular oscillations with lower and slightly out-of-phase recurrence peaks. In Figure 4b, we further plot the polyadic survival probabilities of the two |1, 0⟩ states, which clearly show that the oblique state practically never leaves the polyad, while the local state occasionally leaks out of the polyad. The oblique |1, 0⟩ state evolves coherently within the polyadic block and does so with a recurrence period of 0.257 fs, which coincides technically with that obtained for an initial state constructed as a superposition of the polyadic stationary states ψ_2_ and ψ_3_, given by τ_rec._ = 2.257/(*E*_3_ – *E*_2_) = 0.257 fs. The time evolution of the nonstationary state |0, 1⟩ is identical, for symmetry reasons, to that of the |1, 0⟩ state just described.

**Figure 5:**
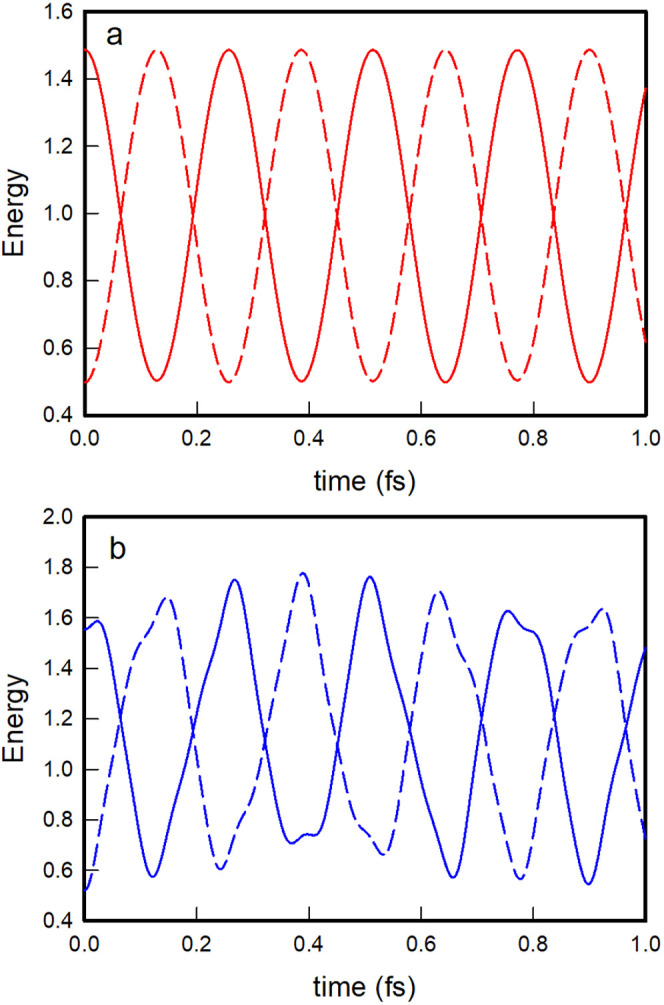
Time evolution of the *E*_1_(*t*) (solid line) and *E*_2_(*t*) (dashed line) energies of the vibrational modes for the oblique (a) and local (b) |1, 0⟩ nonstationary states.

As for the energy content of the vibrational modes, the initial energies deposited in the vibrational mode of the oblique |1, 0⟩ nonstationary state are *E*_1_(0) = ε_1_ = 1.4864 and *E*_2_(0) = ε_2_ = 0.4979, and they evolve with time (eq 58; Figure 5a) in a perfectly regular and periodic way that accounts for a complete exchange of energy between the oblique modes in a period of time equal to the recurrence time of 0.257 fs. For the local |1, 0⟩nonstationary state, the initial energy contents of the vibrational modes are *E*_1_(0) = ε_1_ = 1.5545 and *E*_2_(0) = ε_2_ = 0.5202, and their evolution, although somewhat periodic as well, as observed in Figure 5b, is irregular and abrupt, with some extra energy losses and gains of the vibrational modes due to non-negligible couplings of the local nonstationary state |1, 0⟩ with local states belonging to polyadic groups other than *m* = 1.

Let us consider next the time evolution of the nonstationary states of the polyadic *m* = 2 block. This block is formed by the basis functions |2, 0⟩, |1, 1⟩, and |0, 2⟩, which result to be expressed as the following linear combinations of the stationary states of the complete system in oblique coordinates
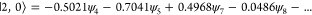
64
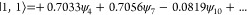
65
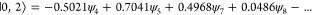
66with energies *E*_|2,0⟩_ = *E*_|0,2⟩_ = 2.9598 and *E*_|1,1⟩_ = 2.9712, and as the following linear combinations in the local coordinates
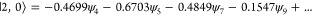
67
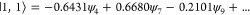
68
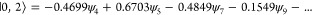
69with energies *E*_|2,0⟩_ = *E*_|0,2⟩_ = 3.0968 and *E*_|1,1⟩_ = 3.1055. The dominant coefficients of the nonstationary states are, both for the oblique and local descriptions, those corresponding to the eigenfunctions ψ_4_, ψ_5_, and ψ_7_ of the Hénon–Heiles system assigned to the *n* = 2 polyadic block (see Table 2). These coefficients are larger in absolute value for the oblique coordinates than for the local coordinates.

**Figure 6:**
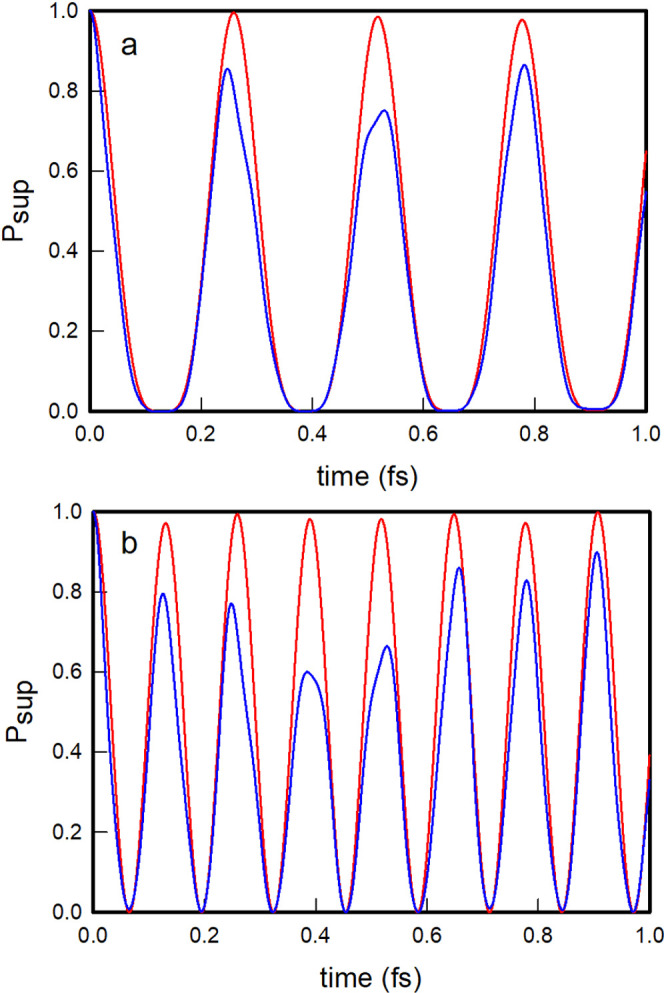
Survival probabilities for the oblique (red) and local (blue) |2,0⟩ (a) and |1, 1⟩ (b) nonstationary states.

In Figures 6a,b, we plot the survival probabilities of the |2, 0⟩ and |1, 1⟩ nonstationary states, respectively, in both oblique and local coordinates. As observed, the oblique states evolve in a more regular oscillatory way than the local states and display noticeably higher recurrence peaks. The recurrence time of the oblique state |2, 0⟩ is 0.259 fs, which is similar to that of the oblique state |1, 0⟩, while the recurrence time of the oblique state |1, 1⟩ roughly halves to 0.130 fs. This value is in practical coincidence with the recurrence time of 2.130/(*E*_7_ – *E*_4_) = 0.130 fs of the nonstationary state formed by a linear combination exclusively of the third and fourth stationary states.

**Figure 7:**
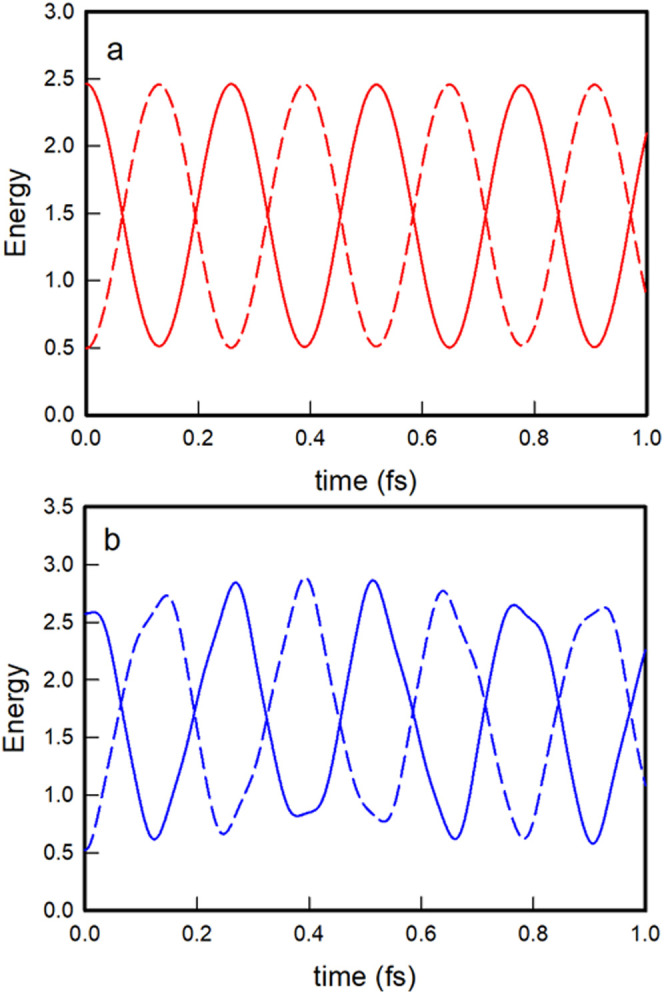
Time evolution of the *E*_1_(*t*) (solid line) and *E*_2_(*t*) (dashed line) energies of the vibrational modes for the oblique (a) and local (b) |2, 0⟩ nonstationary states.

**Figure 8:**
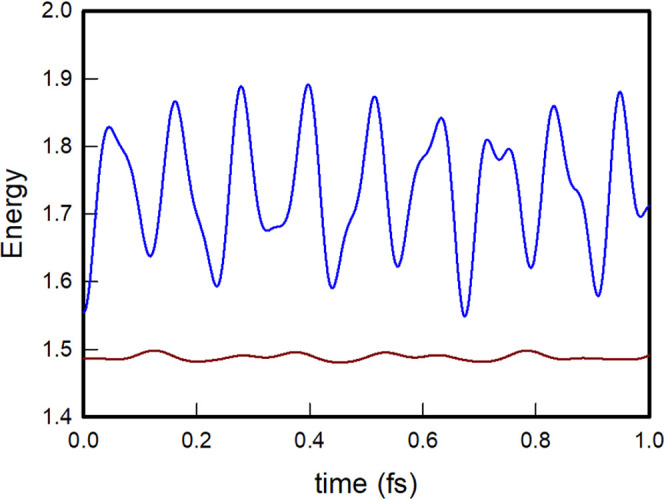
Time evolution of the one-dimensional mode energies of the initial nonstationary state |1, 1,⟩ oblique (red) and local (blue).

As for the energy content of the oscillators, the oblique |2, 0⟩ nonstationary state evolves again by nearly completing regular energy exchanges between the vibrational modes, as shown in Figure 7a, whereas the local |2, 0⟩ state exhibits more abrupt and irregular energy exchanges (Figure 7b). In contrast, the energy behavior of the nonstationary state |1, 1⟩ is completely different. In this case, the two vibrational modes are initially equally excited, each containing one vibrational quantum, and the transition probabilities to the other two nonstationary states within the same poliad, |2, 0⟩ and |0, 2⟩, are equal by symmetry. As a result, the mean values of the energy of each vibrational mode remain the same, with small irregular oscillations due to couplings with states of polyads other than *m* = 2, as shown in Figure 8 for both the oblique and local descriptions. Such irregular oscillations are more pronounced for the local state due to its stronger couplings with states of other polyadic blocks than the oblique state.

The dynamics of the oblique and local nonstationary states of higher polyadic blocks can, in principle, be qualitatively predicted based on what occurs in the polyadic blocks *m* = 1 and *m* = 2, except for any particular effect that may arise when progressively increasing the energy excitation of the vibrational modes. To explore this, we focused on the time evolution of the nonstationary states |*m*_1_, 0⟩, i.e., those initially excited exclusively in the *z*_1_ mode.

**Figure 9:**
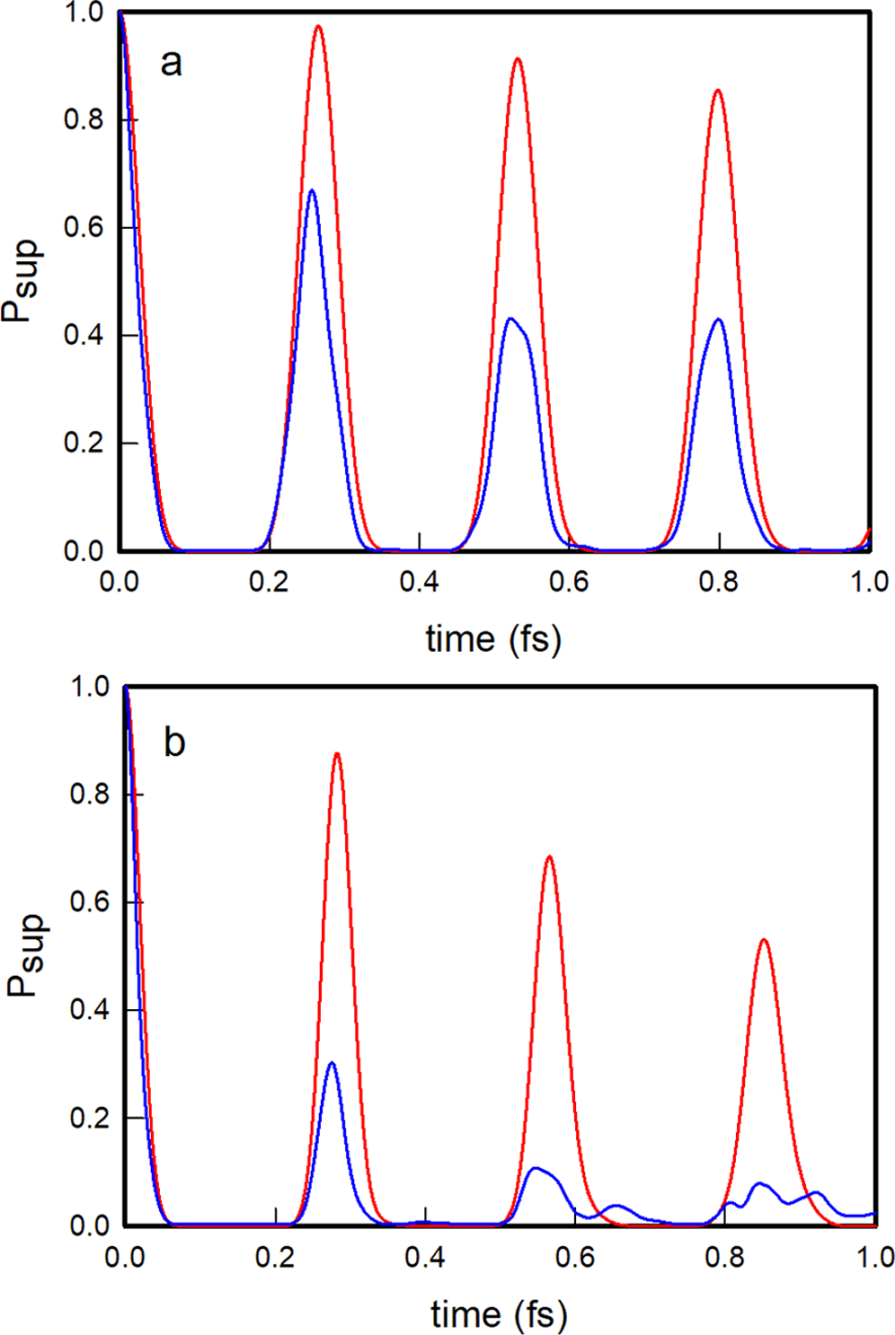
Survival probabilities for the oblique (red) and local (blue) |5, 0⟩ (a) and |10, 0⟩ (b) nonstationary states.

**Figure 10:**
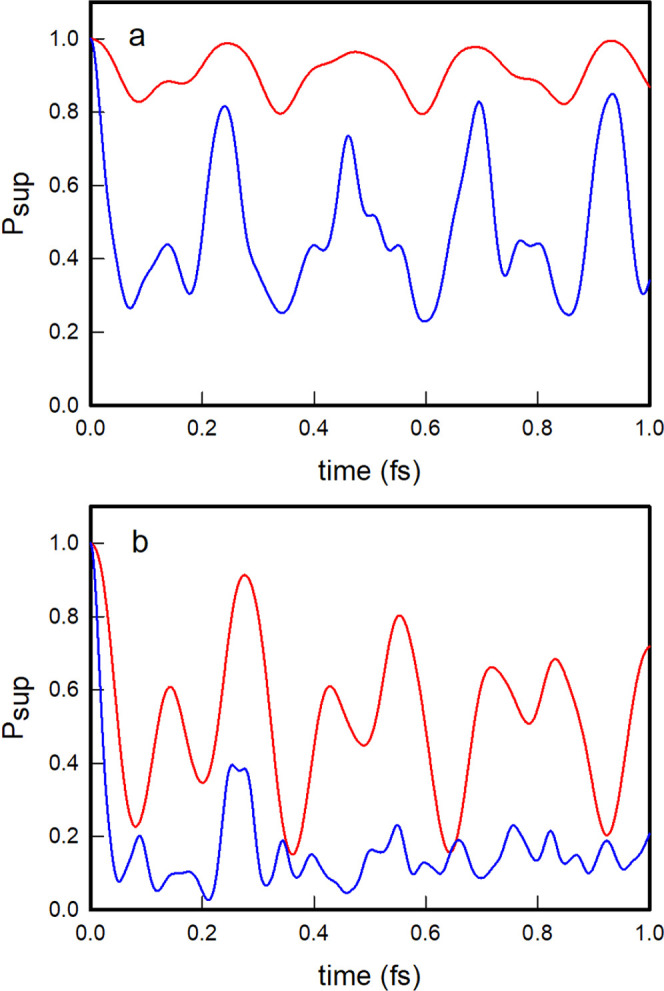
Polyadic survival probabilities for the oblique (red) and local (blue) |5, 0⟩ (a) and |10, 0⟩ (b) nonstationary states.

In Figure 9a, we show the survival probabilities of the |5, 0⟩ oblique and local nonstationary states located in the medium-energy regime of the potential, with energies 5.8065 and 6.0994, respectively, and in Figure 9b, we show the time evolution of the survival probabilities of the |10, 0⟩ oblique and local nonstationary states, with energies of 10.1980 and 10.8029, respectively, very close to the dissociation limit of 11.4601. As expected, the recurrence peaks decrease in height as time progresses but remain appreciably high for the oblique nonstationary states and certainly much higher than the recurrence peaks of the local nonstationary states, which, for the most excited |10.0⟩ state, fade rapidly. Furthermore, the polyadic survival probabilities remain higher for the oblique states than for the local states, as shown in Figure 10a,b. The medium-energy oblique state |5, 0⟩ continues to remain most of the time within its polyadic block, while the oblique state near the dissociation limit |10, 0⟩ clearly explores other polyads, albeit developing significant polyadic recurrences, in contrast to the corresponding local state that dissipates rapidly into other polyads.

In Table 3, we provide a comparison of the energies of all nonstationary |*m*_1_, 0⟩ oblique and local states, along with their first recurrence times and survival probabilities. As observed, the first recurrence times generally become longer as the excitation of the state increases. However, the first recurrence peaks of oblique states remain high, with values above 0.8, even in the most excited states, while the recurrences of local states decay rapidly with increasing excitation.

**Table 3:** Energies and Recurrence Probabilities of the Nonstationary States of the Hénon–Heiles System

	oblique			local		
state	energy	τ_rec._(fs)	*P*_sup_ (τ_rec_)	energy	τ_rec._ (fs)	*P*_sup_ (τ_rec_)
|1, 0⟩	1.9839	0.257	0.9985	2.0736	0.243	0.9154
|2, 0⟩	2.9598	0.259	0.9953	3.0968	0.247	0.8557
|3, 0⟩	3.9228	0.261	0.9860	4.1091	0.251	0.7951
|4,0⟩	4.8721	0.263	0.9828	5.1095	0.254	0.7328
|5, 0⟩	5.8065	0.265	0.9738	6.0994	0.257	0.6687
|6, 0⟩	6.7249	0.268	0.9636	7.0757	0.260	0.6028
|7, 0⟩	7.6255	0.271	0.9520	8.0381	0.264	0.5351
|8, 0⟩	8.5066	0.274	0.9379	8.9851	0.268	0.4642
|9, 0⟩	9.3655	0.278	0.9171	9.9134	0.272	0.3846
|10, 0⟩	10.1980	0.282	0.8768	10.8029	0.275	0.3034
|11, 0⟩	10.9866	0.287	0.7894			

**Figure 11:**
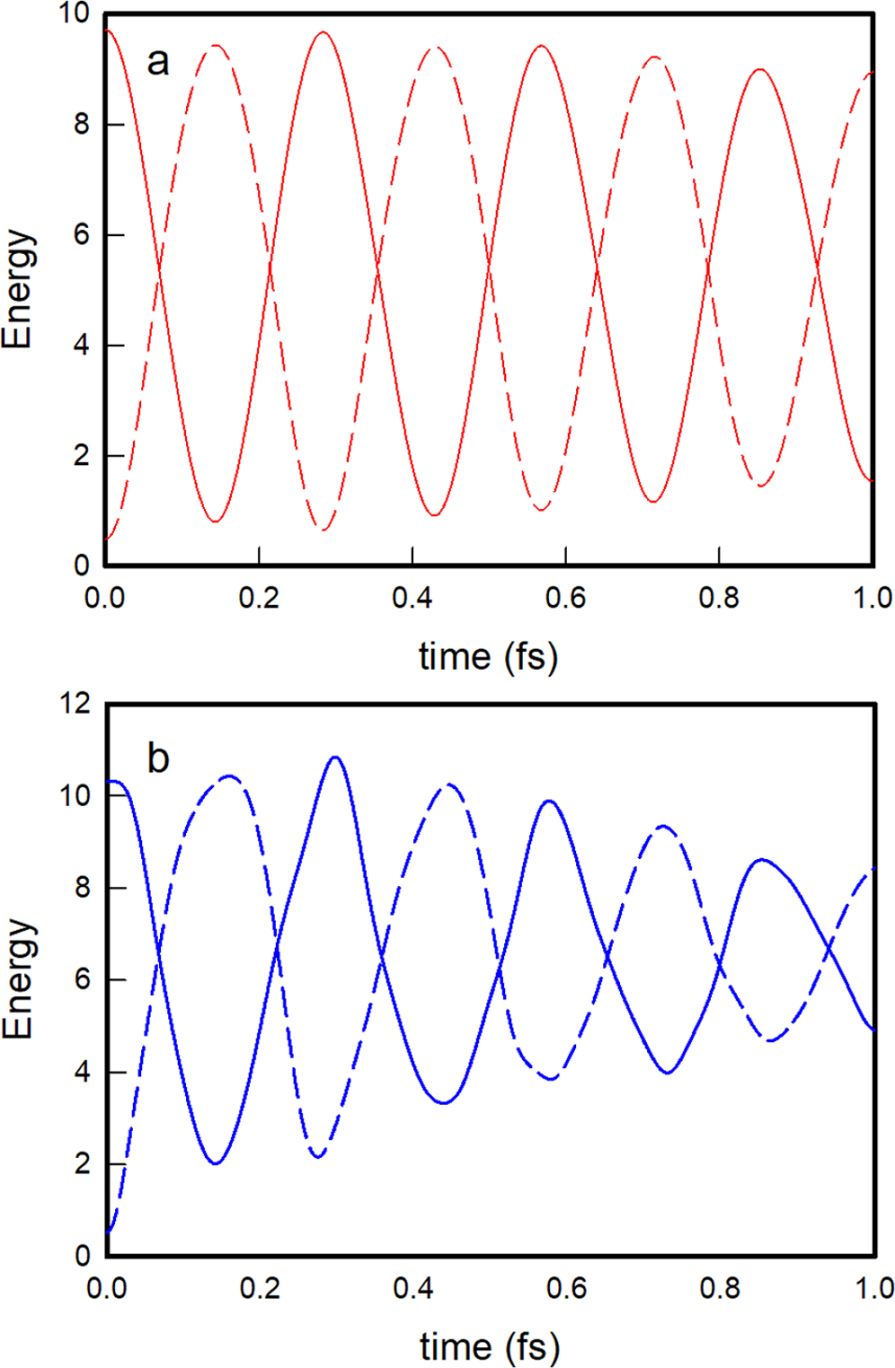
Time evolution of the *E*_1_(*t*) (solid trace) and *E*_2_(*t*) (dashed trace) energies of the one-dimensional modes of the initial nonstationary states |10, 0⟩ oblique (a), and local (b).

Finally, as far as the energy content of the vibrational modes is concerned, the energy exchange between the oblique modes remains quite regular and complete, even in the most excited nonstationary states such as |10, 0⟩, as shown in Figure 11a. In the local nonstationary state |10, 0⟩, the exchange is, as we already know, somewhat rough and, more importantly, less efficient as time goes on, as evidenced by the gradual decrease of the amplitudes of the energy oscillations seen in Figure 11b.

## 4 Conclusions

In this work, we have comparatively studied the quantum dynamics of oblique nonstationary states versus rotated, or local, nonstationary states in a system of coupled Hénon–Heiles oscillators with two dissociation channels that can be used to model the vibrational stretching motions of symmetric triatomic molecules. The local nonstationary states are the eigenstates of the zero-order Hamiltonian operator obtained by performing a 45° orthogonal rotation of the normal coordinates, while the oblique nonstationary states are the zero-order states obtained by performing a nonorthogonal coordinate rotation that allows us to write the matrix representation of the second-order Hamiltonian in a block diagonal form characterized by the polyadic quantum number *n* = *n*_1_ + *n*_2_.

First, we performed variational calculations of all of the bound vibrational states of the Hénon–Heiles system up to the dissociation limit using the three coordinate systems, normal, local, and oblique, and confirmed that the vibrational spectrum is practically regular over the entire range of bound energies. We have also shown that the oblique coordinates enable us to assign all states to their corresponding polyadic groups, as opposed to the local coordinates, whose polyadic assignments start to fail from the middle energy region upward.

As a consequence of the oblique coordinates’ efficiency in organizing the stationary states of the Hénon–Heiles system into polyadic blocks with *n* = *n*_1_ + *n*_2_, we have demonstrated that the time evolution of oblique nonstationary states is much more periodic and coherent than that of the corresponding local nonstationary states, as evidenced by both the survival probabilities and the energy exchange between vibrational modes. The quantum time dynamics of nonstationary oblique states occur mainly within the polyadic group to which the initial excited state belongs. Furthermore, the time dynamics of such states remain quite regular up to the dissociation limit on a short time scale, as demonstrated by the well-defined profiles of the recurrence peaks and the energy exchange between the oblique vibrational modes occurring in a regular, oscillating manner with little loss.

In view of our recent work on the usefulness of oblique coordinates in two-dimensional systems, both in the time-independent^33−36^ and time-dependent (present work) approaches, it is clear that the next step is to extend oblique coordinates to systems with a larger number of degrees of freedom such as the vibrational motions taking place in polyatomic molecules and in condensed phase systems. One of the advantages of oblique coordinates over normal coordinates is the greater number of parameters available in the former, which is useful to optimize them conveniently with greater flexibility. However, it is to be expected that as the dimensionality increases, the possibility of obtaining closed analytical expressions for the optimal values of the oblique parameters will quickly fade away, and numerical methods will have to be resorted to, as occurs with normal coordinates. All this opens, in our opinion, a wide range of possibilities for using oblique coordinates in systems with three or more degrees of freedom, both to construct highly separable oblique coordinate systems that computationally facilitate the variational determination of vibrational states and their physical interpretation, as well as to design multidimensional oblique nonstationary states that propagate with a high degree of coherence, facilitating the transfer of energy between vibrational modes in a more selective way. Logically, our future work on oblique coordinates points in all of these directions.
